# Semi-supervised method for image texture classification of pituitary tumors via CycleGAN and optimized feature extraction

**DOI:** 10.1186/s12911-020-01230-x

**Published:** 2020-09-09

**Authors:** Hong Zhu, Qianhao Fang, Yihe Huang, Kai Xu

**Affiliations:** 1 School of Medical Information, Xuzhou Medical University, Xuzhou, China; 2 Department of Computer Science and Engineering, State University of New York at Buffalo, Buffalo, USA; 3 Affiliated Hospital of Xuzhou Medical University, Xuzhou, China

**Keywords:** Pituitary tumors, CycleGAN, DenseNet, ResNet, Auto-encoder, CRNN

## Abstract

**Background:**

Accurately determining the softness level of pituitary tumors preoperatively by using their image textures can provide a basis for surgical options and prognosis. Existing methods for this problem require manual intervention, which could hinder the efficiency and accuracy considerably.

**Methods:**

We present an automatic method for diagnosing the texture of pituitary tumors using unbalanced sequence image data. Firstly, for the small sample problem in our pituitary tumor MRI image dataset where T1 and T2 sequence data are unbalanced (due to data missing) and under-sampled, our method uses a CycleGAN (Cycle-Consistent Adversarial Networks) model for domain conversion to obtain fully sampled MRI spatial sequence. Then, it uses a DenseNet (Densely Connected Convolutional Networks)-ResNet(Deep Residual Networks) based Autoencoder framework to optimize the feature extraction process for pituitary tumor image data. Finally, to take advantage of sequence data, it uses a CRNN (Convolutional Recurrent Neural Network) model to classify pituitary tumors based on their predicted softness levels.

**Results:**

Experiments show that our method is the best in terms of efficiency and accuracy (91.78%) compared to other methods.

**Conclusions:**

We propose a semi-supervised method for grading pituitary tumor texture. This method can accurately determine the softness level of pituitary tumors, which provides convenience for surgical selection and prognosis, and improves the diagnostic efficiency of pituitary tumors.

## Background

Pituitary tumor is one of the most common diseases in the nervous system [1]. It is the third largest tumor type in brain and is extremely harmful to the human body [2]. Many critical questions, such as whether a surgical procedure is needed, what kind of procedure is most suitable, and what is the expected postoperative effect, are all closely related to the softness of pituitary tumor [3]. It is important to accurately judge the softness level of pituitary tumor preoperatively in a non-invasive manner. This has been a problem for a long time and is still plaguing the clinic. However, due to the closure nature of the cranial cavity, it is often difficult to accurately determine the softness of pituitary tumor before surgery [4]. With the technological advancements in medical imaging, MR, CT and other imaging modality can now provide rich anatomical information non-invasively. It has been shown that such information can be used to improve the treatment planning for 30 to 50% cancer patients, resulting in more accurate treatments for them. Thus, it is extremely valuable to mine deep quantitative information (such as the softness level) from pituitary tumor image data, which is not perceivable by the naked eyes of clinician.

At present, the most commonly used method for evaluating the image texture of pituitary tumor is image omics, which is defined as the conversion of visual image information into deep features for quantitative research. The advantage of such a method is its interpretability, which is based on domain knowledge [5]. Aerts et al [6] extracted 440 CT features for prognosis, and found that imaging histology can reflect tumor phenotype, internal heterogeneity, and the prognostic radiological features of intra-tumoral heterogeneity are related to potential gene expression patterns, which could effectively assess the prognosis of patients. Zhang et al [7] adopted an approach that combines machine learning techniques with imaging omics. They extracted 970 medical image features, and used six kinds of machine learning phenomenological feature selection methods and nine classification methods to obtain 54 different combinations. They showed that the random forest method (RF) has the best performance in the prognosis analysis of nasopharyngeal carcinoma images. However, since image omics requires accurate extraction of lesions, it is not very efficient. Moreover, the number of deep features that can be extracted by image omics could be as many as thousands, which need to be selected manually. Thus, it is a challenging task to select the best set of features, as it depends largely on the experience of the technician. In general, feature extraction is a computation-intensive and time-consuming process, and thus better solution is needed.

In recent years, artificial intelligence has gained a lot of popularity which propelled a new way for medical imaging processing. The combination of deep learning and medical imaging. It has shown that such an approach is capable of automatically extracting a large number of deep features from large medical image datasets, and yields much improved solutions. For example, Wang et al. [8] combined medical imaging with in-depth learning to develop a new generation of image reconstruction theory technology, which enhanced the ability of image analysis and image reconstruction. Xu et al. [9] proposed a new network cxnet-m1 to detect abnormal chest X-ray images, which improved the efficiency and accuracy of diagnosis. Wei et al. [10] proposed a method called Locality-constrained Sparse Autoencoder (LSAE) which introduces the concept of locality into Autoencoder and can encode similar inputs by similar features. Their method achieves a classification accuracy of 72.7% for CALTECH-101 dataset. Xu et al. [11] presented a new Stacked Sparse Autoencoder (SSAE) framework for the diagnosis of high resolution histopathological images of breast cancer. They used a dataset with 500 histopathological images (2200 × 2200) and 3500 manually segmented cell nucleuses, and showed that their method improves the F value by 84.49% and yields an AVEP of 78.83%.

Despite the aforementioned progresses, deep-learning-based approaches are also facing a number of challenges, such as data unbalancing in small sample, limited reliably labeled data, inaccurate feature extraction, etc. In the case of pituitary tumor, the dataset we collected is unbalanced, e.g., only T1 sequences but lacking of T2 sequences. In addition, more accurate features of pituitary tumor image data are needed for texture classification. In this paper, we proposed a semi-supervised pituitary tumor image classification method based on CycleGAN and optimized feature extraction. Our method first uses CycleGAN to make up the missing T2 sequences, and then adopts a DenseNet-ResNet based Autoencoder-decoder framework to extract pituitary tumor features and optimize adaptively. Finally, the optimized features are inputted to CRNN. It needs only sequence-level label, instead of frame-level label, to complete the training for subtype classification of pituitary tumors.

## Methods

### Theoretical basis

#### CycleGAN

CycleGAN [12] is basically two mirrored GANs that form a ring network. The goal of CycleGAN is to convert image A to another domain to generate image A1 and convert A1 back to A, where output image A1 is similar to the original input image A to form a meaningful mapping that does not exist in the unpaired data set. The advantage of CycleGAN is its ability to train two image sets without pairing.

#### DenseNet

DenseNet [13] is a convolutional neural network framework with dense connectivity proposed by Huang Gaoren in 2017. In its architecture, there is a direct connection between any two layers of the network. The input of each layer of the network is a combination of the output of all previous network layers, which enhances the propagation of features. It alleviates the problem of gradient disappearance, reduces network parameters and encourages feature reuse. It has been widely used in the medical image field.

#### ResNet

ResNet [14] is a convolutional neural network framework proposed by He et al. in 2015. It adds a shortcut on top of the original architecture to enable direct connection between the mappings of layers, which solves the degradation problem. ResNet alleviates the gradient vanishing and gradient explosion problems caused by the increased depth of the network, and thus protects the entirety of the data. It has been widely used in medical image field.

#### CRNN

CRNN is a model proposed by Shi et al. [15] to deal with sequence-like objects in images, which consists of DCNN and RNN. DCNN is used to extract sequence features from the input image. RNN has the advantage of processing sequence data, and can achieve better recognition accuracy from the extracted sequence features. The ability of CRNN to predict sequence data brings inspiration to the recognition of medical image data.

### Pituitary tumor sequence data amplification using CycleGAN

A problem often encountered in MR images of pituitary tumors is under-sampling in a single domain (e.g., T1 or T2). This can be caused by various reasons, such as data missing or simply under sampling. To resolve this issue, our main idea is to use images from other domains (which may come from different image modalities) to generate a set of new images through domain conversion. The set of new and old images forms an augmented set of images which provides a better sample for the domain.

Particularly, we use CycleGAN for data augmentation. First, two domain converters are designed and trained based on the CycleGAN architecture to allow inter-domain conversion from T1 to T2 and from T2 to T1. Then, the generated MR images from domain conversion are added to the original sets of images to form augmented T1 and T2 sequences.

#### Multiple sequence of pituitary tumor MR images

As mentioned above, the MR images of one patient usually include spatial sequences from different modalities, such as T1WI, T2WI, T1C and T2FLAIR, etc. In this paper, we mainly use T1 and T2 spatial sequence images.

For each patient i, we denote its T1 spatial sequence is as ${\mathrm{T}}_{1\mathrm{i}}=\left\{{t}_{1,1}^i,\dots, {t}_{1,N}^i\right\}$_,_ where ${t}_{1,n}^i$ represents the n-th slice/frame in the T1 spatial sequence, and its T2 spatial sequence as ${\mathrm{T}}_{2\mathrm{i}}=\left\{{t}_{2,1}^i,\dots, {t}_{2,N}^i\right\}$, where ${t}_{2,n}^i$ represents the n-th slice/frame in the T2 spatial sequence. The number of slices per sequence is N (12 in this paper). To classify the pituitary tumors, we combine the T1 and T2 spatial sequences of each patient i to obtain a spatial sequence of multiple sequences, which is denoted as:

1 $$ {\mathrm{T}}_{\mathrm{i}}=\left\{\left({t}_{1,1}^i,{t}_{2,1}^i\right),\left({t}_{1,2}^i,{t}_{2,2}^i\right).\dots, \left({t}_{1,N}^i,{t}_{2,N}^i\right)\right\} $$

The total number of slices in a multi-sequence spatial sequence is 2 N (24 in this paper).

#### Training domain converter based on CycleGAN

**Fig. 1:**
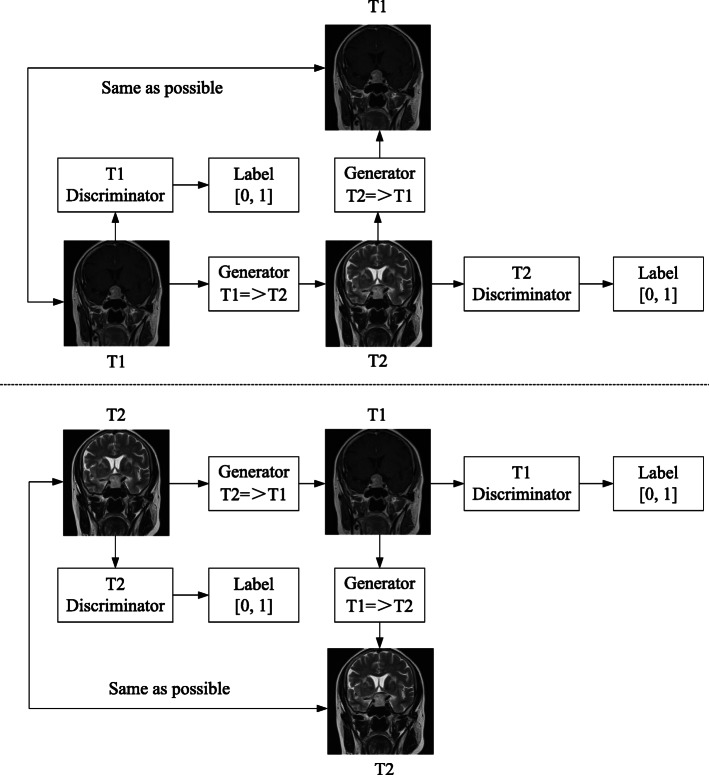
CycleGAN based Deep Neural Network Model for domain conversion

In this paper, we use the CycleGAN framework to design and train the domain converter. CycleGAN is essentially a cyclic network consisting of two mutually symmetric GANs. On top of the original GAN, additional loop constraints are added to force the image to be converted into its original image format so as to reconstruct itself. This allows images to be converted from one domain to another domain without needing to pair them. The architecture of our domain converter is illustrated in Fig. 1. In our design, we need to train the T1-to-T2 generator Tr(t_1, n_; θ_1_) and the T2-to-T1 generator Tr(t_2, n_; θ_2_), as well as the T1 domain discriminator Dis(t_1, n_; θ_3_) and T2 domain discriminator Dis(t_2, n_; θ_4_), where in θ_1_, θ_2_, θ_3_ and θ_4_ are the to-be-determined parameters in the deep neural network. During the training process, when the discriminator’s loss reaches the minimum and tends to be stable, CycleGAN model training is completed.

**Fig. 2:**
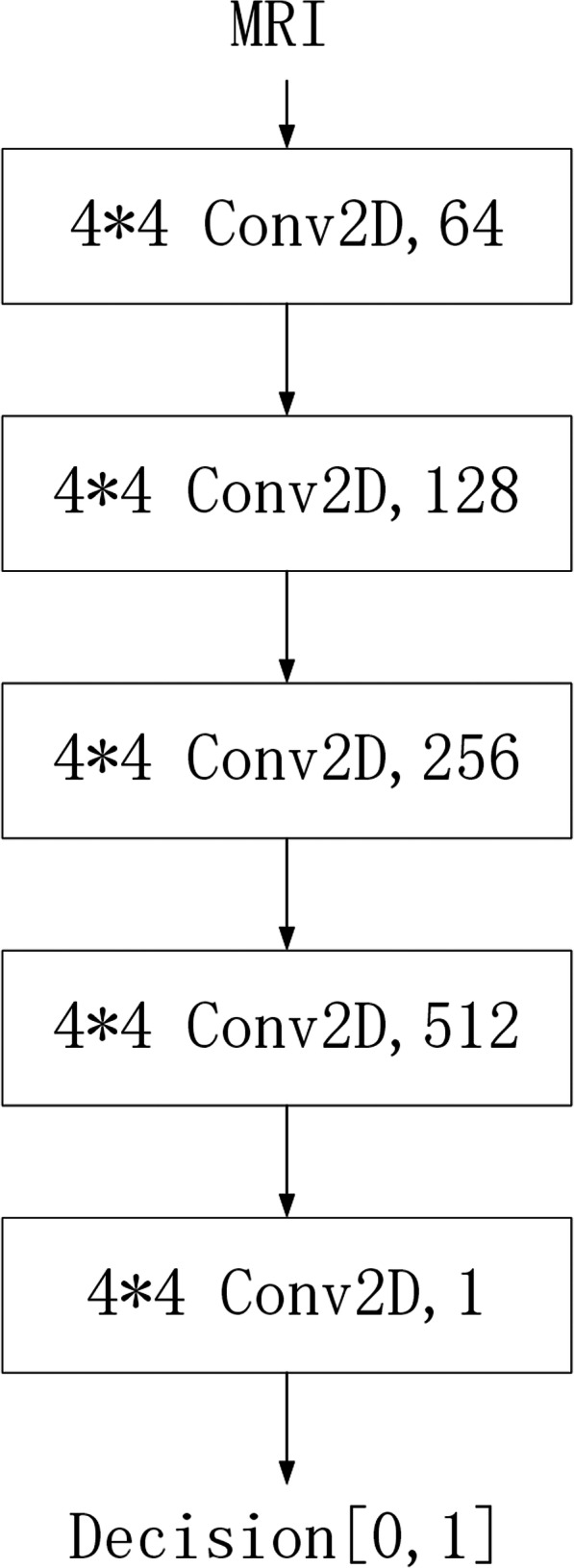
Discriminator architecture

**Fig. 3:**
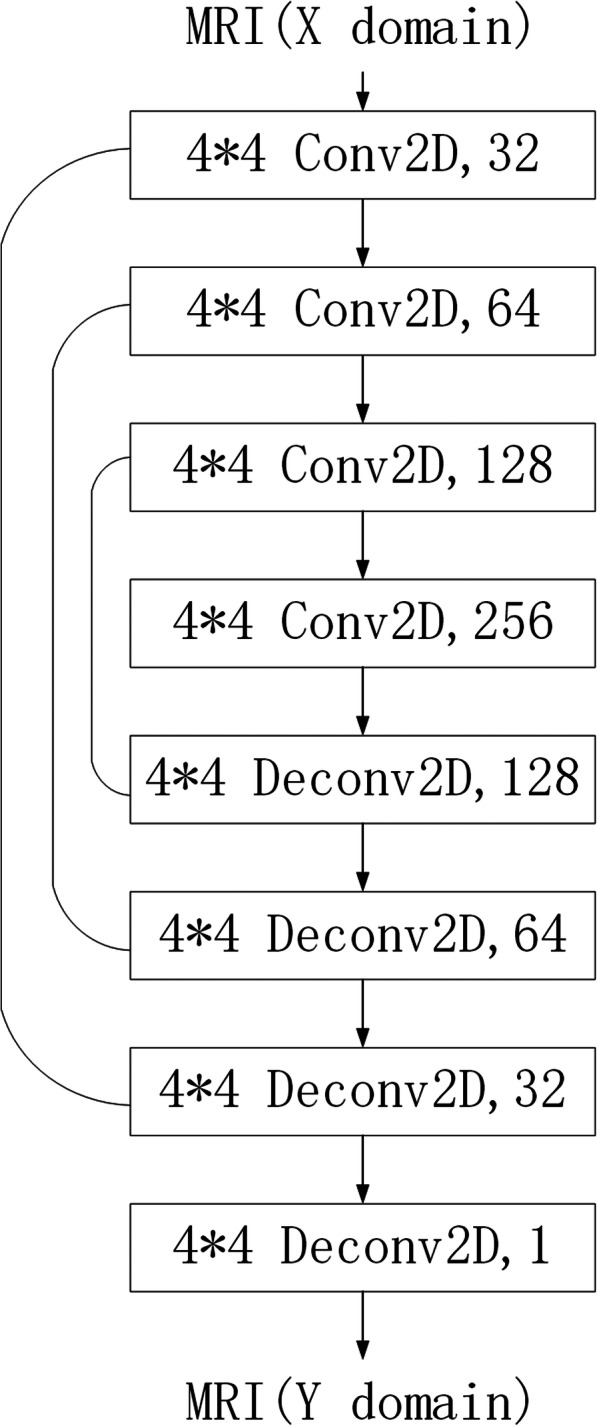
Generator architecture

The training of the above network mainly consists of two steps:

1. The training of the Discriminator: Fixing the values of θ_1_ and θ_2_, update the values of θ_3_ and θ_4_. This is for discriminating the authenticity of the image. If the input is MR image data from the real domain, the label is 1, and if the input is an MR image data generated by the generator, the label is 0. In short, the role of the discriminator is to score pictures. If the input pictures are real pictures from the original dataset, they will get high scores. Otherwise (i.e., they are generated fake pictures), their scores will be low. The network of this part is depicted in Fig. 2, where the convolution layer is consists of Conv2D, Leaky ReLU, Instance Normalization, and the digits represent the size and number of the convolution kernel.
2. The training of the Generator: Fixing the values of θ_3_ and θ_4_, update the values of θ_1_ and θ_2_. This is for inter-domain conversion of images. After getting the input MR images from T1 or the T2 domain, the generator sends them to the corresponding domain converter to generate MR images of the other domain. The generated images are then again sent to the corresponding domain converter to generate MR images of the original domain. After being converted twice, the obtained MR images are forced to be as similar as possible to the original ones. The network of this part is shown in Fig. 3, where the convolution layer consists of Conv2D, Leaky ReLU, Instance Normalization. The first three de-convolution layers consist of UpSampling2D, Conv2D, ReLU, Instance Normalization, and the last de-convolution layer consists of UpSampling2D, Conv2D, Tanh. The dashed line represents the superimposing operation between the corresponding network layers, and the digits are the size and number of the convolution kernel.

### Semi-supervised classification method for the image texture of pituitary tumors based on adaptively optimized feature extraction

To improve the efficiency of feature extraction for determining the softness level of pituitary tumor, using DenseNet, ResNet we propose in this paper an Auto-Encoder-based deep neural network model for feature extraction. Since the weight of the features common to all input data could be reduced during the training process, our proposed model can enhance the weight of the features unique to each MRI spatial sequence (i.e., the features of pituitary tumor), and meanwhile reduce the dimensionality of the features of each slice. This can greatly accelerate the operational speed of the subsequent classifier. Therefore, it is essential for our classification method to use the proposed Auto-Encoder-based framework for feature extraction.

#### Encoder and decoder based on dense block and residual block

**Fig. 4:**
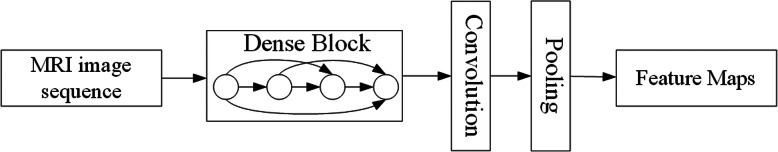
ResNet encoder network architecture

For encoder, we use Dense Block to enhance the feature propagation ability of MRI spatial sequences, rely on the convolutional layer and pooling layer to reduce the dimensionality, and combine them to form an encoder for extracting the common features of MRI spatial sequences. As shown in Fig. 4, the encoder uses two dense blocks in the training process (only one is shown in the figure). Due to the fact that the feature maps are superimposed during the training process, it enhances the propagation ability of pituitary tumor features, which consequently improves the accuracy and reliability of feature extraction.

**Fig. 5:**
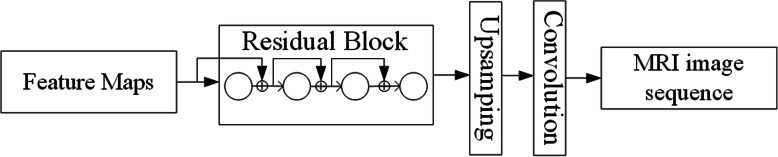
ResNet decoder network architecture

For decoder, we use Residual Block to compress the dimensionality of the feature map, and rely on the upsampling layer and the convolution layer to increase the dimensionality. These two components together form the decoder which can generate MRI spatial sequences with the same dimensionality as the original input data. The network architecture is shown in Fig. 5. It also uses two residual blocks in the decoder (only one is shown in the figure). The decoder uses shortcut to lower the weight of some features during the training process. Also, the model drift increases due to the added network depth (after adding the decoder). These together improve the effectiveness of MRI spatial sequence reconstruction. It also means that it is quite meaningful to use Residual Block for image reconstruction in the whole model.

#### Adaptive optimization of feature extraction

**Fig. 6:**
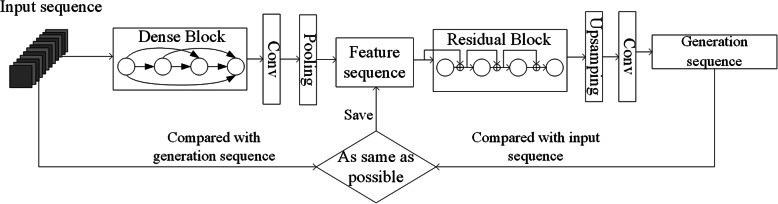
Feature extraction model based on Auto-Encoder

The above Dense Block-based encoder and Residual Block-based decoder enable us to adopt an Auto-Encoder model for sequence level feature extraction. Its specific network structure is shown in Fig. 6.

Firstly, the input image sequence is generated by dense block encoder. Secondly, the feature sequence is decoded by the residual block-based decoder to restore the image sequence. At last, the input image is compared with the corresponding pixels of the generated image. The lower the loss, the more similar the generated image is to the input image, and the more representative the extracted feature sequence is.

#### Semi-supervised classification of spatial sequence images based on CRNN

**Fig. 7:**
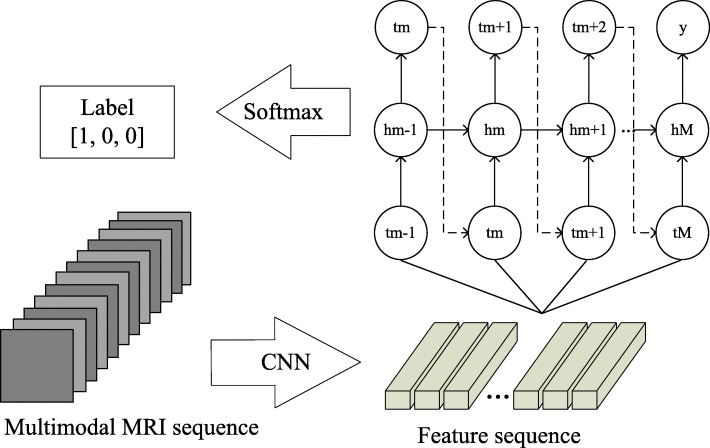
CRNN classifier network architecture

The extracted feature map of the pituitary tumor MRI spatial sequence is a three-dimensional matrix using the format of CRNN. An image sequence represents a patient and only one sequence-level label is needed. We first use CNN to extract the spatial feature sequence of the feature map, and then use RNN to train the extracted feature sequence. When the loss in training process reaches the lowest and tends to be stable, it indicates that CRNN model has been trained. At this time, the model can be used as a standard to predict test accuracy. The neural network architecture is shown in Fig. 7.

#### Multi-sequence pituitary tumor classification model

**Fig. 8:**
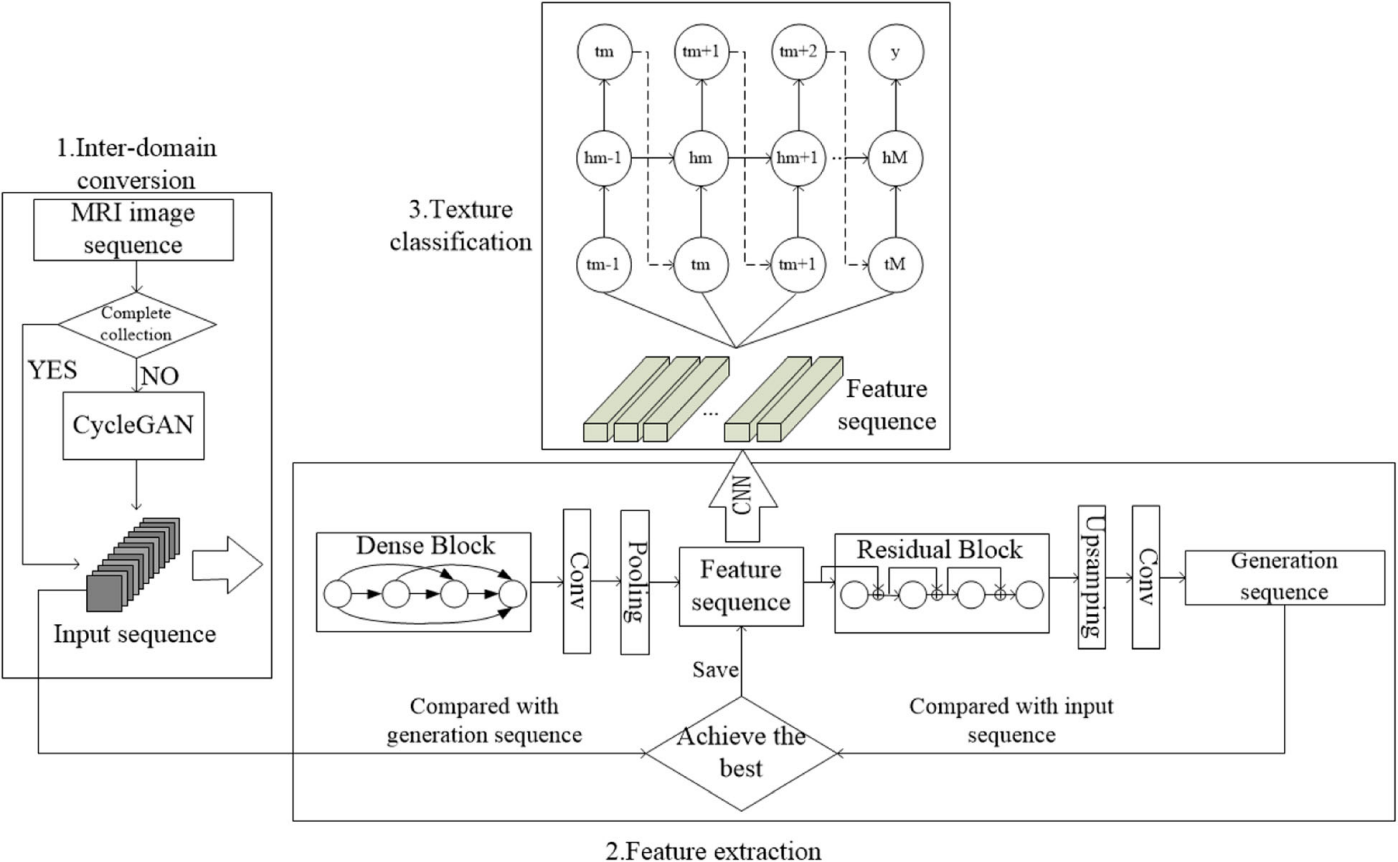
Multi-sequence pituitary tumor grading model

Combining all the above neural network components, we obtain a model for classifying the multi-sequences of pituitary tumors. Its network architecture is shown in Fig. 8. The model is capable of augmenting under-sampled T1 and T2 datasets, fusing sequences from multiple modalities, extracting features, and finally obtaining the accurate estimation of the softness level of pituitary tumor by using a CRNN-based classifier.

### Experiment platform and dataset

Our experiments are conducted in the following settings. The operating system is Windows10, the processor is 2.10GHz Intel Xeon (dual core), the memory capacity is 64GB, the development environment is PyCharm, the deep learning framework is Keras, the programming language is Python, and the graphics card is GeForce RTX 2080Ti (three cores).

The dataset used in the experiment was pituitary tumors collected in a local affiliated hospital. Each patient had MRI data of OAX, OSAG and OCOR (In this paper, OCOR MRI data are used), and there were T1 and T2 two modes in OCOR MRI data. There are 374 patients in total, 152 of whom are labeled, with each associated with a grading label from the following two grades: soft texture and hard texture.

## Results

### Experiment analysis

#### CycleGAN-based multi-sequence data amplification

**Fig. 9:**
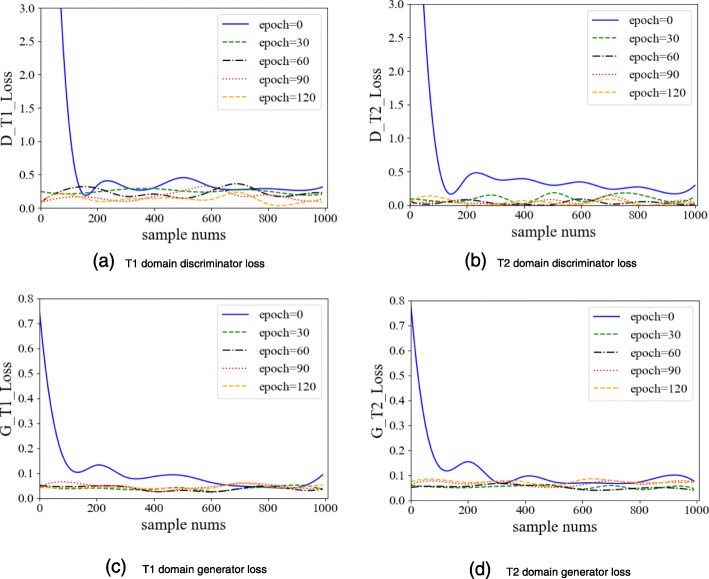
Discriminator loss and generator loss

We use the image data of 374 patients for CycleGAN training, including 280 T1 MRI spatial sequences and 94 T2 MRI spatial sequences. We train a total of 120 times, in which the loss of the generator and the discriminator is shown in Fig. 9. When the number of training reaches 90 epochs, the loss of the discriminator reaches its minimum and becomes stable.

**Fig. 10:**
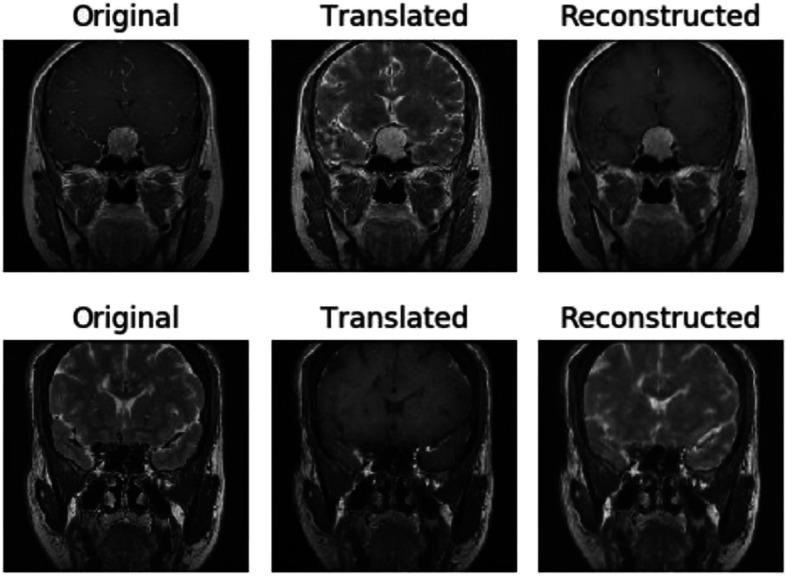
Visualization of training results

We use 152 patient datawith labels (including 112 T1 MRI spatial sequences and 40 T2 MRI spatial sequences) to augment the data using the trained cyclegan model. As a result, there is a multi-sequence of 24 slices (12 T1 slices and 12 T2 slices) for each patient. The result (after 120 times of training) is shown in Fig. 10.

Figure 10 shows the original MR image in two domains and the MR image reconstructed after two conversions by the domain converter. Visually, the difference between a real MR image and a transformed MR image is very small.

#### Semi-supervised pituitary tumor texture image classification based on adaptively optimized feature extraction

After being amplified by CycleGAN, the dataset was then fed to the Auto-Encoder for feature extraction using unsupervised learning. Supervised learning is conducted during the CRNN texture classification stage.

**Fig. 11:**
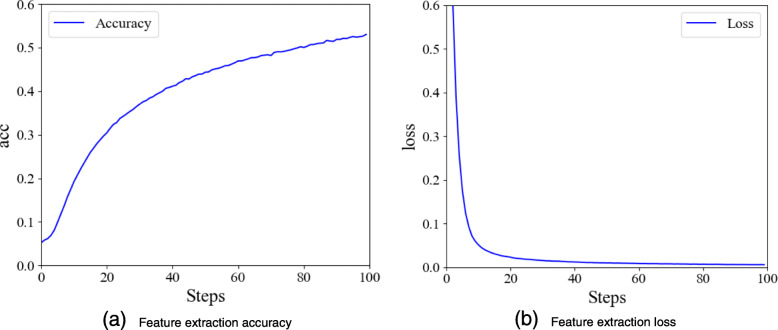
Multi-sequence feature extraction model

To ensure reliable comparisons, all the models were trained 100 steps in the feature extraction stage. The training process of multi-sequences is shown in Fig. 11, and the curve of the single-modal baseline is similar.

It can be seen from the figure that when the model is trained 100 steps, the loss curve reaches its lowest point, which is 0.01, and feature extraction network almost achieves the optimal solution.

The architecture of the experiment can be divided into three models, namely the multi-sequence model, the T1 domain model and the T2 domain model. The multi-sequence (medical image classification) model is compared to two single-modal baseline models:

1. T1 domain model: We only consider the MRI spatial sequence of T1 domain of all patients, including the MRI spatial sequence generated from another domain converter.
2. T2 domain model: We only consider the MRI spatial sequence of T2 domain of all patients, including the MRI spatial sequence generated from another domain converter.
3. Multi-sequence model: We use the trained domain converter to construct an MRI multi-sequence in both T1 and T2 domains, including the MRI spatial sequence generated by the domain converters.

**Fig. 12:**
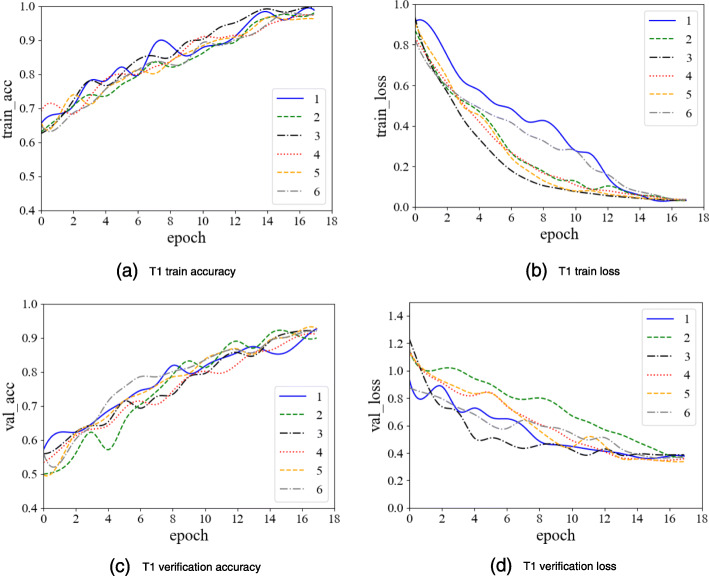
T1 domain image classification model training

**Fig. 13:**
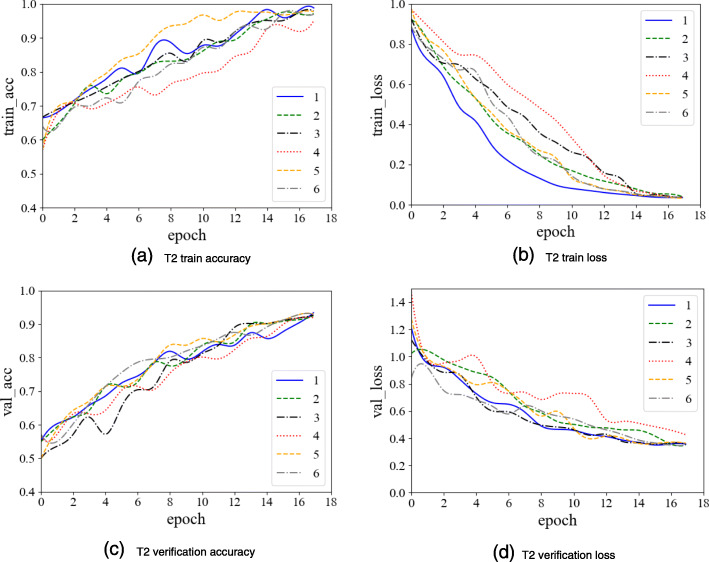
T2 domain image classification model training

**Fig. 14:**
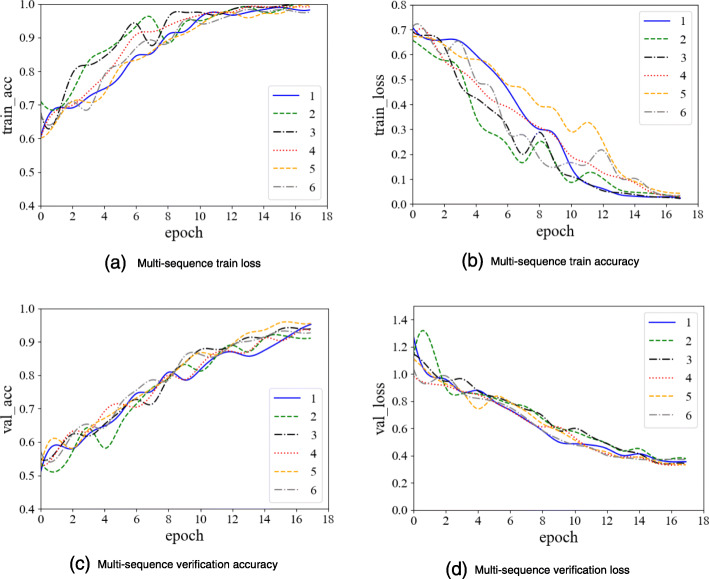
Multi-sequence image classification model training

In the texture classification stage, there are many neural network model parameters in the experiment, but a small number of trained samples. This could potentially cause over-fitting. To avoid this issue, we use Dropout and EarlyStopping methods during the training process. The Droupout ratio is set to be 0.5, that is, for all the neural network units in model, they are temporarily discarded from the network with a probability of 50%. We set the patience value of EarlyStopping to be 2 and the monitor to be ‘val_loss’. That is, if the value of ‘val_loss’ does not decrease relative to the previous epoch during model training, the model is stopped after 2 epochs. The T1 domain, T2 domain, and multi-sequence model training process are shown in the following figures:

As can be seen from Figs. 12, 13, 14, we performed 6 replicate experiments on the T1 domain, T2 domain and the multi-sequence domain. In our experiment, we randomly divide the dataset into training dataset (70%), test dataset (15%), and verification dataset (15%). We repeated this process 6 times, and recorded the average and variance of 6 classification accuracy rates. Table 1 shows the details of classification, and Table 2 shows precision, recall and F1-score of classification:

**Table 1:** Pituitary tumor classification accuracy

	Multi-sequence(%)	T1 domain(%)	T2 domain(%)
Train	98.8 ± 1.24	97.55 ± 1.40	97.41 ± 1.37
Verification	92.82 ± 1.23	91.70 ± 1.61	91.15 ± 1.13
Test	91.78 ± 1.44	89.24 ± 3.11	88.98 ± 4.23

**Table 2:** Precision, recall and F1-score of pituitary tumor classification

	Precision(%)	Recall(%)	F1-score(%)
T1 domain	86.81 ± 3.67	93.33 ± 5.96	89.80 ± 2.64
T2 domain	87.07 ± 3.71	94.44 ± 5.02	90.41 ± 2.15
Multi-sequence	89.89 ± 4.02	95.55 ± 5.44	92.46 ± 1.74

#### Compared with other models

In the training process of neural network model, the quality of tumor feature extraction is the key to improving the classification accuracy. Compared with the feature extraction method of image omics, deep learning can adaptively learn the tumor features from big data. Table 3 lists the performance of ours and some commonly used classification models on pituitary tumor MRI images. This further demonstrates the effectiveness of our method.

**Table 3:** Comparisons of classification results of different methods

Feature extraction	Texture classification	Accuracy(%)	Time(s)
–	VGG	69	113
–	ResNet	78.25	105
–	DenseNet	81.25	97
–	CRNN	73.7	67
ResNet+ ResNet	CRNN	88.76	43
DenseNet+DenseNet	CRNN	90.33	43
DenseNet+ResNet	CRNN	91.78	42
DenseNet+ResNet	RNN	89.12	42

As can be seen from the above table, our proposed DenseNet+ResNet+CRNN architecture significantly outperforms all other methods in terms of running time and classification accuracy. Our method has the fastest convergence rate and thus shortest running time. From the perspective of classification accuracy, we can see that adding an Auto-Encoder-based feature extractor before CRNN can considerably improve the performance. In summary, the comparative experiment suggests that our CycleGAN-based classification model and the adaptively optimized feature extraction has great potential of yielding accurate texture classification results for pituitary tumors.

In order to verify the clinical statistical significance of the experiment, we paired the method proposed in this article with the other methods in Table 3. We use Wilcoxon signed rank test to perform statistical test on paired samples, and the specific data are shown in Table 4.

**Table 4:** Statistics of Wilcoxon signed rank test based on paired samples

Feature extraction	Texture classification	Z	*P*
–	VGG	−2.201	0.028
–	ResNet	−2.201	0.028
–	DenseNet	−2.201	0.028
–	CRNN	−2.201	0.028
ResNet+ ResNet	CRNN	−2.201	0.028
DenseNet+DenseNet	CRNN	−2.023	0.043
DenseNet+ResNet	RNN	−2.201	0.028
DenseNet+ResNet	CRNN	–	–

It can be seen from Table 4 that the *P* values obtained by statistics on various models are all less than 0.05, which is statistically significant. Results have clinical significance.

**Fig. 15:**
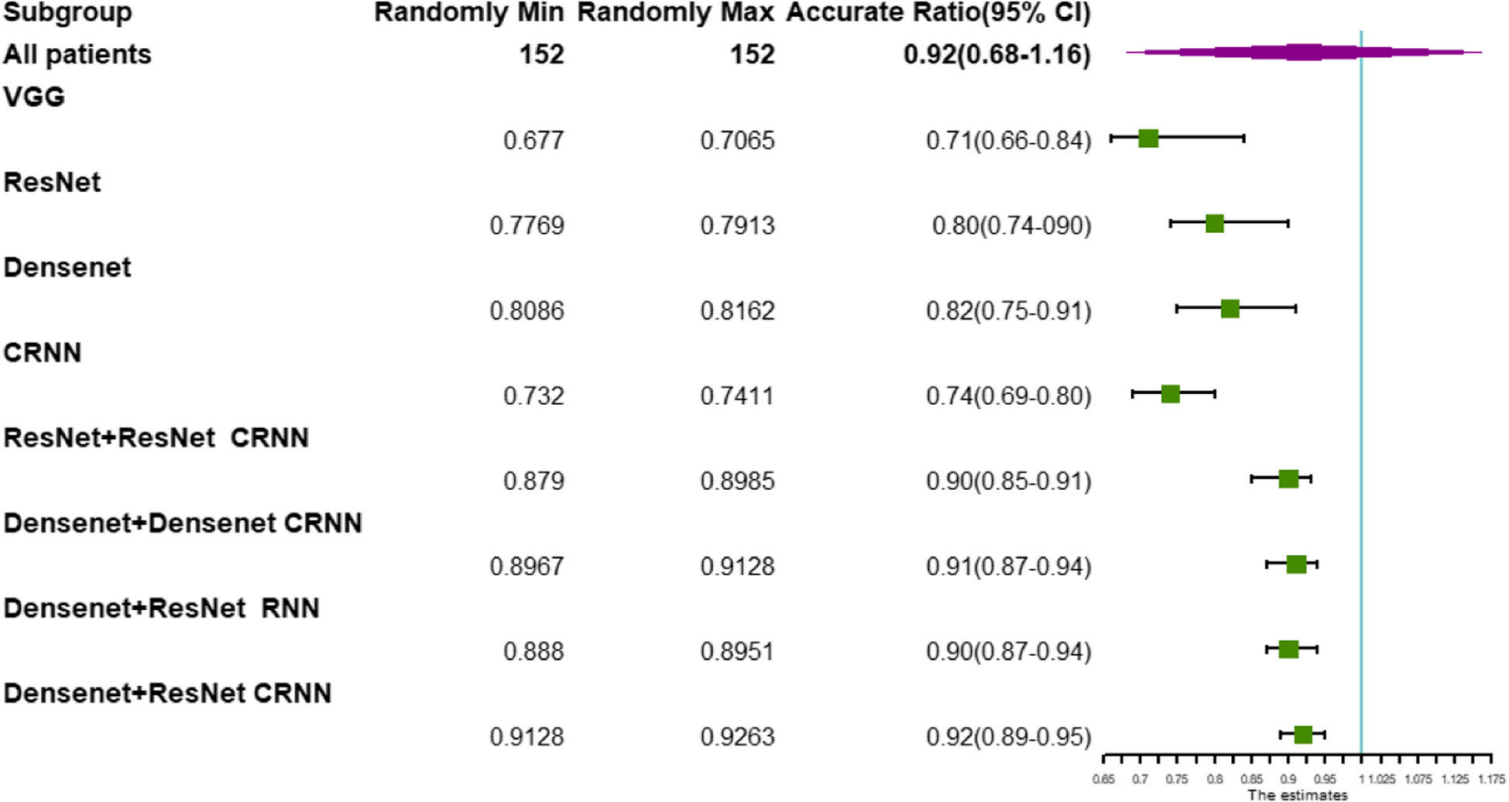
Forest plot for comparisons of classification results of different methods

In order to reflect this contrast more clearly, we have drawn a forest plot, as shown in Fig. 15.

As can be seen from the forest plot, our proposed method is more effective compared with other methods.

## Discussion

In this study, several experiments were designed to validate our method. Particularly, We first carried out a comprehensive evaluation of the image data generated by CycleGAN, and found that the generated images were great. Subsequently, we list the training curves of feature extraction part to judge extraction effect. Finally, we repeated the experiment six times, calculated the test accuracy, and compared it with other models, and found that our method is the best in terms of accuracy and efficiency. These experiments demonstrate that our method has advantages in grading pituitary tumors. Despite the achievements reported in this paper, several improvements remain possible: On the one hand, the data samples used in the experiment are still insufficient, and it is easy to produce the phenomenon of overfitting. On the other hand, although the loss of feature extraction model training is low and convergence is achieved, the accuracy is still not high enough. Future research in the domain shall address these issues, possibly collecting new data and improving the part of feature extraction.

## Conclusion

In this paper, we proposed a deep neural network model for determining the softness level of pituitary tumors, which has the potential to assist clinical diagnosis. Our method first uses CycleGAN to amplify the pituitary tumor dataset to generate multi-sequence samples, which enhances the diversity of pituitary tumor samples and thus helps resolve the under-sampling issue. Then, our method uses an Auto-Encoder architecture, based on ResNet encoding and decoding, to extract the pituitary tumor features, which can improve the classification efficiency of the network to some extent. Finally, the extracted pituitary tumor features are fed to CRNN for classification/grading of the softness level of pituitary tumors. Experiments on a real medical dataset show that our method achieves significantly improved results than some existing popular methods. The experimental results also suggest that our adaptively optimized feature extraction method can better identify deep texture features of pituitary tumor image, and can thus improve the classification accuracy of pituitary tumors.

## Data Availability

The data that support the findings of this study are available from [Kai Xu] but restrictions apply to the availability of these data, which were used under license for the current study, and so are not publicly available. Data are however available from the authors upon reasonable request and with permission of [Kai Xu].

## Abbreviations

CycleGAN: Cycle-Consistent Adversarial Networks
DenseNet: Densely Connected Convolutional Networks
ResNet: Deep Residual Networks
CRNN: Convolutional Recurrent Neural Network
